# Clinical outcomes of uni-portal non-coaxial spinal endoscopic surgery versus unilateral biportal endoscopic surgery for lumbar spinal stenosis: a retrospective cohort study

**DOI:** 10.3389/fsurg.2026.1780210

**Published:** 2026-05-20

**Authors:** Xiao Min Cai, Guo Sen Li, Xiao Yang Nie, Da Liu, Chun Lin Xiao, Min Zhao, En Song, Jiang Jun Zhou

**Affiliations:** 1 Department of Orthopaedics, The 908th Hospital of Joint Logistic Support Force, Nanchang, Jiangxi, China; 2 Third Clinical Medical College of Nanchang University, Jiangxi Medical College, Nanchang University, Nanchang, Jiangxi, China; 3 Department of Orthopaedics, The General Hospital of Western Theater Command, Chengdu, Sichuan, China; 4 Department of Orthopaedics, The First Affiliated Hospital of Gannan Medical University, Ganzhou, Jiangxi, China; 5 Department of Orthopaedics, The People’s Hospital of Yingtan City, Yingtan, Jiangxi, China; 6 Department of Orthopaedics, First Affiliated Hospital of Kunming Medical University, Kunming, China

**Keywords:** endoscopic decompression, lumbar spinal stenosis, minimally invasive surgery, unilateral biportal endoscopy, uni-portal non-coaxial spinal endoscopic surgery

## Abstract

**Objective:**

To compare the clinical efficacy and safety of uni-portal non-coaxial spinal endoscopic surgery (UNSES) with unilateral biportal endoscopic technique (UBE) for the treatment of lumbar spinal stenosis (LSS).

**Methods:**

We retrospectively analyzed 195 patients with LSS (96 in the UNSES group and 99 in the UBE group) from January 2022 to December 2023. The two groups were compared regarding operative time, postoperative hemoglobin decrease, incision length, length of hospital stay, complication rate, functional scores (VAS, JOA, ODI), and imaging parameters, including dural sac cross-sectional area (DCSA), lumbar range of motion (ROM), and sagittal translation (ST).

**Results:**

No significant differences were observed in baseline characteristics between the UNSES and UBE groups. Compared with the UBE group, the UNSES group showed shorter operative time (65.2 ± 8.1 vs. 72.5 ± 9.3 min, *P* < 0.001), lower hemoglobin decrease (6.2 ± 1.4 vs. 6.8 ± 2.5 g/L, *P* = 0.039), a smaller incision length (1.8 ± 0.3 vs. 2.5 ± 0.4 cm, *P* < 0.001), and fewer fluoroscopy exposures (3.1 ± 1.2 vs. 4.5 ± 2.0, *P* < 0.001). Both groups showed postoperative improvement in pain, functional scores, and DCSA compared with preoperative values (*P* < 0.05), whereas ROM and ST remained unchanged. No intergroup differences were observed in these parameters or in complication rates at any time point (all *P* > 0.05).

**Conclusion:**

At the 1-year follow-up, UNSES and UBE were associated with comparable clinical and radiographic outcomes in patients with single-segment lumbar spinal stenosis. Compared with UBE, UNSES was associated with differences in several perioperative parameters, including operative time, hemoglobin decrease, incision length, and fluoroscopy exposure. These findings should be interpreted with caution, given the retrospective design and limited follow-up duration.

## 1 Introduction

Lumbar spinal stenosis (LSS) is a common spinal disorder caused by narrowing of the spinal canal that compresses neural and vascular structures. Degenerative changes, including ligamentum flavum hypertrophy, facet joint osteoarthritis, and intervertebral disc degeneration, are the main etiological factors (1), leading to symptoms such as lower-extremity pain, numbness, and weakness (2). The estimated mean prevalence of degenerative LSS, based on clinical diagnoses, ranges from 11% to 39%, while estimates based on radiological diagnoses range from 11% to 38% (3). LSS is the most common indication for spinal surgery in individuals over 65 years of age (4).

Although traditional open decompression surgery yields satisfactory clinical outcomes, it is associated with significant structural damage, considerable blood loss, delayed recovery, and residual low back pain (5). The advent of minimally invasive techniques has opened new avenues for LSS treatment. Unilateral biportal endoscopy (UBE) achieves comprehensive visual decompression of the spinal canal and nerve roots through the synergistic action of independent observation and working channels. Clinical studies have confirmed that UBE is comparable to open surgery in efficacy while significantly reducing the incidence of postoperative lumbar instability (6, 7). A 5-year follow-up study reported an excellent UBE rate of 90.6%, with no significant changes in dynamic stability (8). However, the dual-incision design of UBE results in an average of 5.79 ± 1.24 intraoperative x-ray exposures per procedure (9), and mastering the technique requires a learning curve of at least 54 cases (10). Heo et al. also reported that, although UBE has a longer learning curve than uniportal endoscopy and microsurgery, it can achieve efficient decompression once mastered (11). Experience with sports medicine endoscopic techniques may reduce intraoperative fluoroscopy exposures and lower the learning curve.

To expand the repertoire of minimally invasive techniques, uni-portal non-coaxial spinal endoscopic surgery (UNSES) was developed. This technique was first proposed by Professor En Song's team in 2021 to address certain limitations of traditional endoscopic techniques (12). Specifically, UNSES integrates the dual-port approach into a single portal, allowing the endoscope and instruments to operate independently within the same portal while maintaining good maneuverability (13), thereby effectively reducing incision length and intraoperative blood loss (14). However, direct comparative evidence of UNSES vs. UBE remains limited. In this retrospective cohort study, we systematically compared perioperative indices and functional outcomes of the two procedures to inform clinical decision-making.

## 2 Methods

### 2.1 Study design and enrollment criteria

This single-center, retrospective, controlled study included patients with LSS who underwent minimally invasive surgery at our institution's Department of Spine Surgery between January 2022 and December 2023. The study protocol was approved by the Institutional Review Board (IRB) of the 908 Hospital, and written informed consent was obtained from all participants.

Inclusion criteria were: (1) single-segment LSS diagnosed by CT or MRI, with typical neurogenic claudication or radicular symptoms; (2) failure of conservative treatment (medication, physiotherapy, etc.) for at least 12 weeks; (3) American Society of Anesthesiologists (ASA) classification ≤ II, able to tolerate general anesthesia.

Exclusion criteria were: (1) history of lumbar spine surgery or traumatic spinal injury; (2) dynamic radiography suggesting segmental instability (slippage >3 mm or angulation >10°); (3) concomitant spinal deformity, infection, or tumor; (4) pregnancy or serious systemic disease (e.g., coagulopathy).

The final enrollment of 195 patients was divided into two groups based on surgical approach: the UNSES group (*n* = 96) underwent uniportal noncoaxial spinal endoscopic surgery, and the UBE group (*n* = 99) underwent dual-channel endoscopic techniques.

### 2.2 Surgical operation standardization

All procedures were performed by a single, experienced spine surgeon (with over 10 years of experience in minimally invasive spine surgery and having completed the UBE learning curve of 50 cases). Before the study began, the surgeon had performed 50 UNSES cases to overcome the initial learning curve, ensuring that the comparison reflected the technique itself rather than a learning effect. The surgeon also received standardized training in UNSES from the technique developer.

#### 2.2.1 UNSES technique

**Figure 1:**
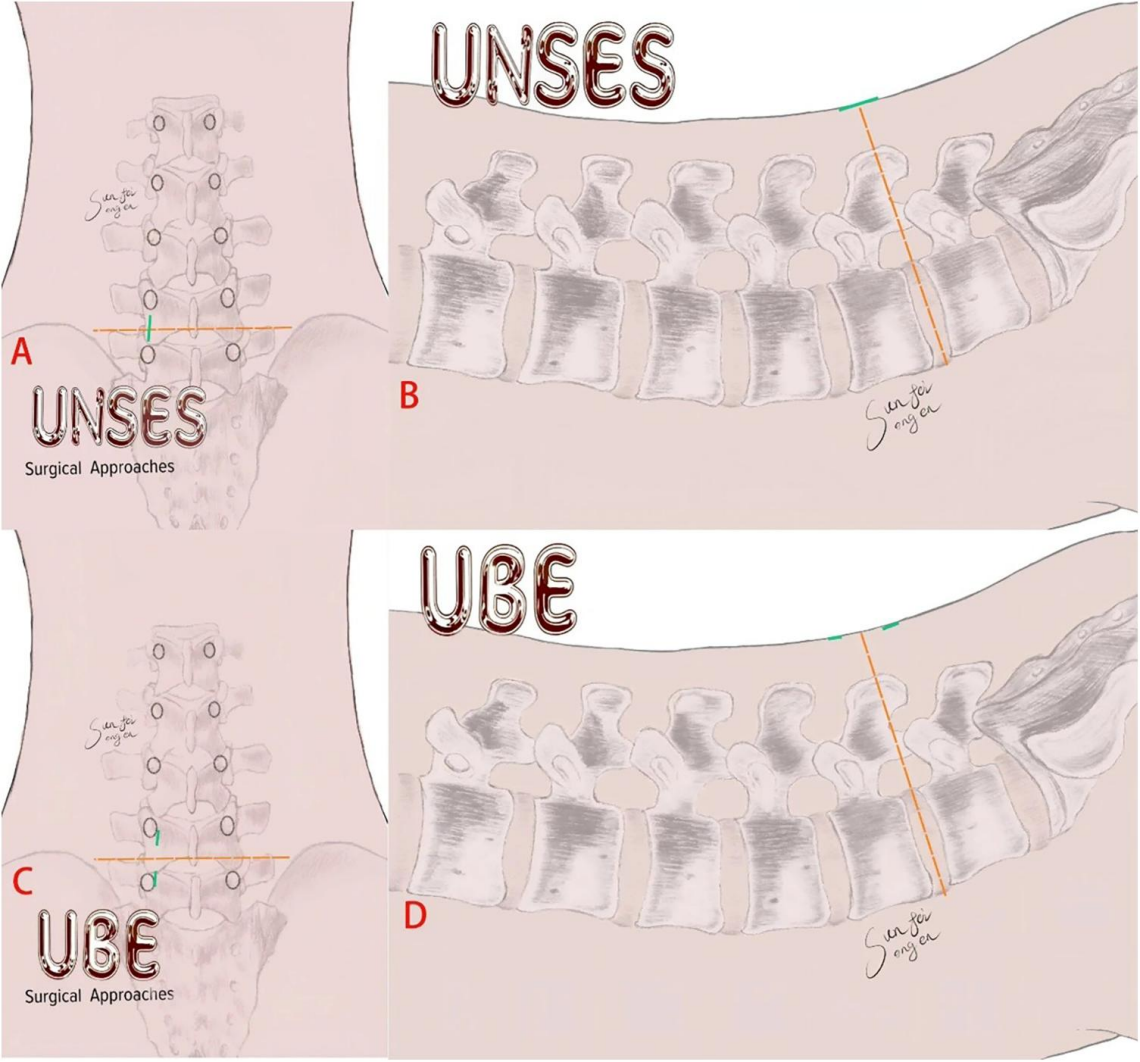
Surgical incision diagram. **(A,B)** Show the UNSES incision; **(C,D)** show the UBE incision (AUSS-UNSES).

**Figure 2:**
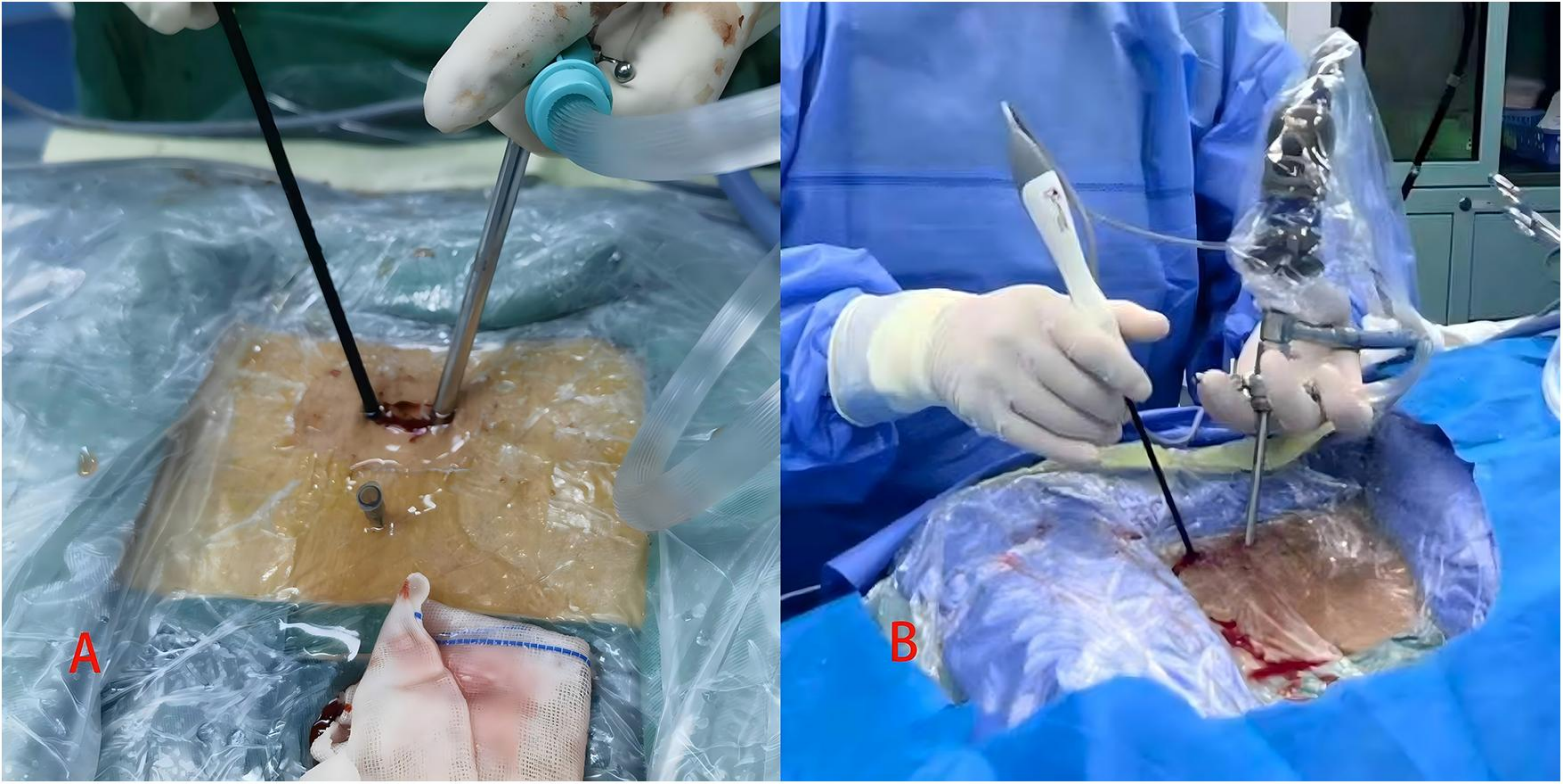
**(A)** Is an intraoperative diagram of the UNSES procedure; **(B)** is an intraoperative diagram of the UBE procedure.

**Figure 3:**
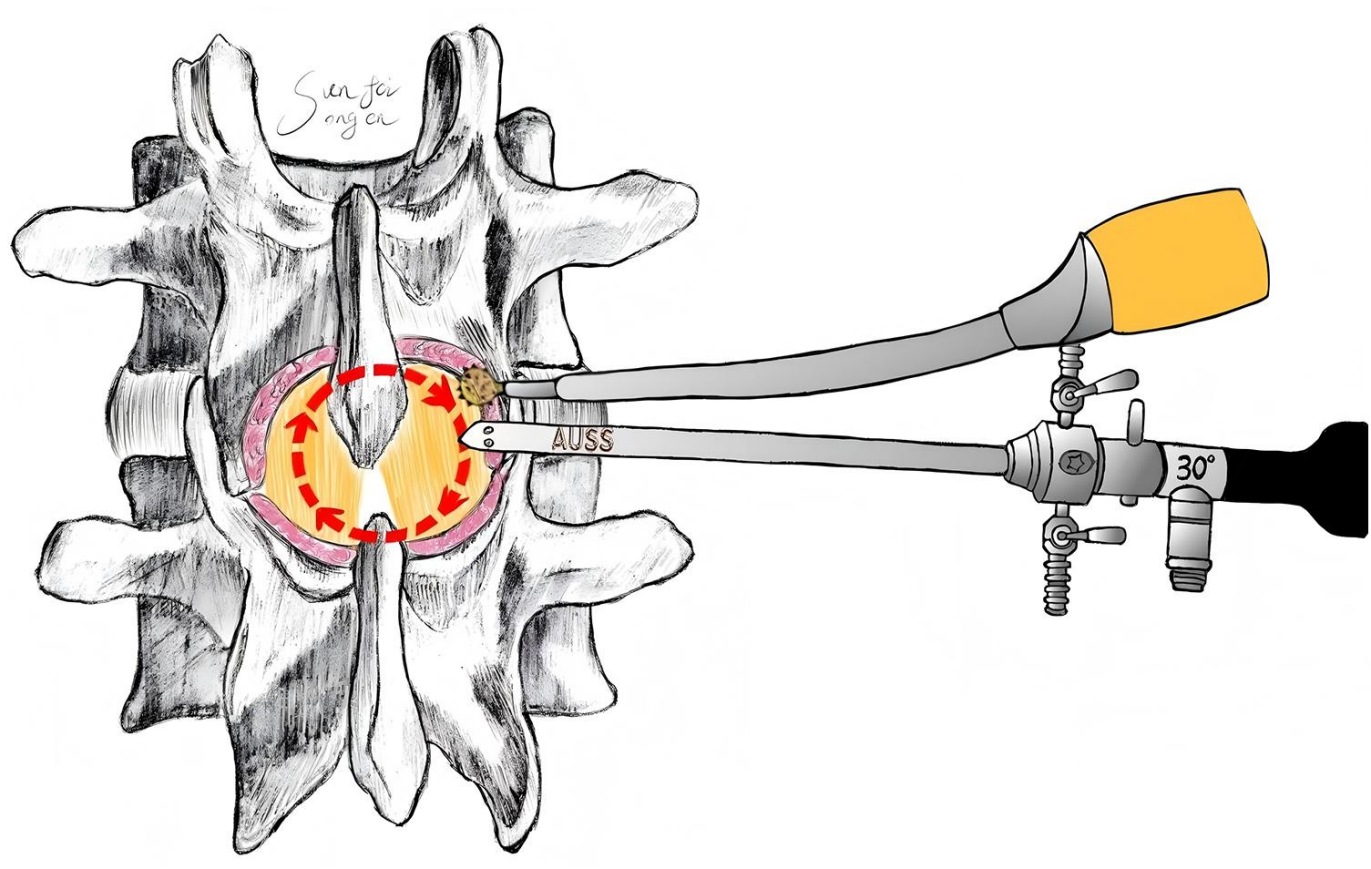
The modified circumferential ULBD technique (“circle-drawing method”) involves sequential exposure and resection: beginning at the spinous process base-lamina junction of the superior vertebra, partial bone resection with a high-speed burr or osteotome reveals the ligamentum flavum insertion site (anatomical cleavage plane). Ipsilateral decompression proceeds by removing bone from the inferior margin of the superior vertebra to the ligamentum flavum insertion, followed by resection of the inferior articular process to expose the ipsilateral facet joint space and superior articular facet tip of the inferior vertebra. Subsequent resection targets the superior margin of the inferior lamina and spinous process base to expose the corresponding ligamentum flavum insertion. Contralaterally, laminar bone is removed in clockwise/counterclockwise directions to expose the ligamentum flavum insertion, facet joint space, and superior articular facet tip of the contralateral inferior vertebra. Finally, the ligamentum flavum is resected either piecemeal or *en bloc* following adhesion exploration and release, with technique selection determined by intraoperative assessment of adhesion severity between the ligamentum flavum and dural sac and surgeon preference.

**Figure 4:**
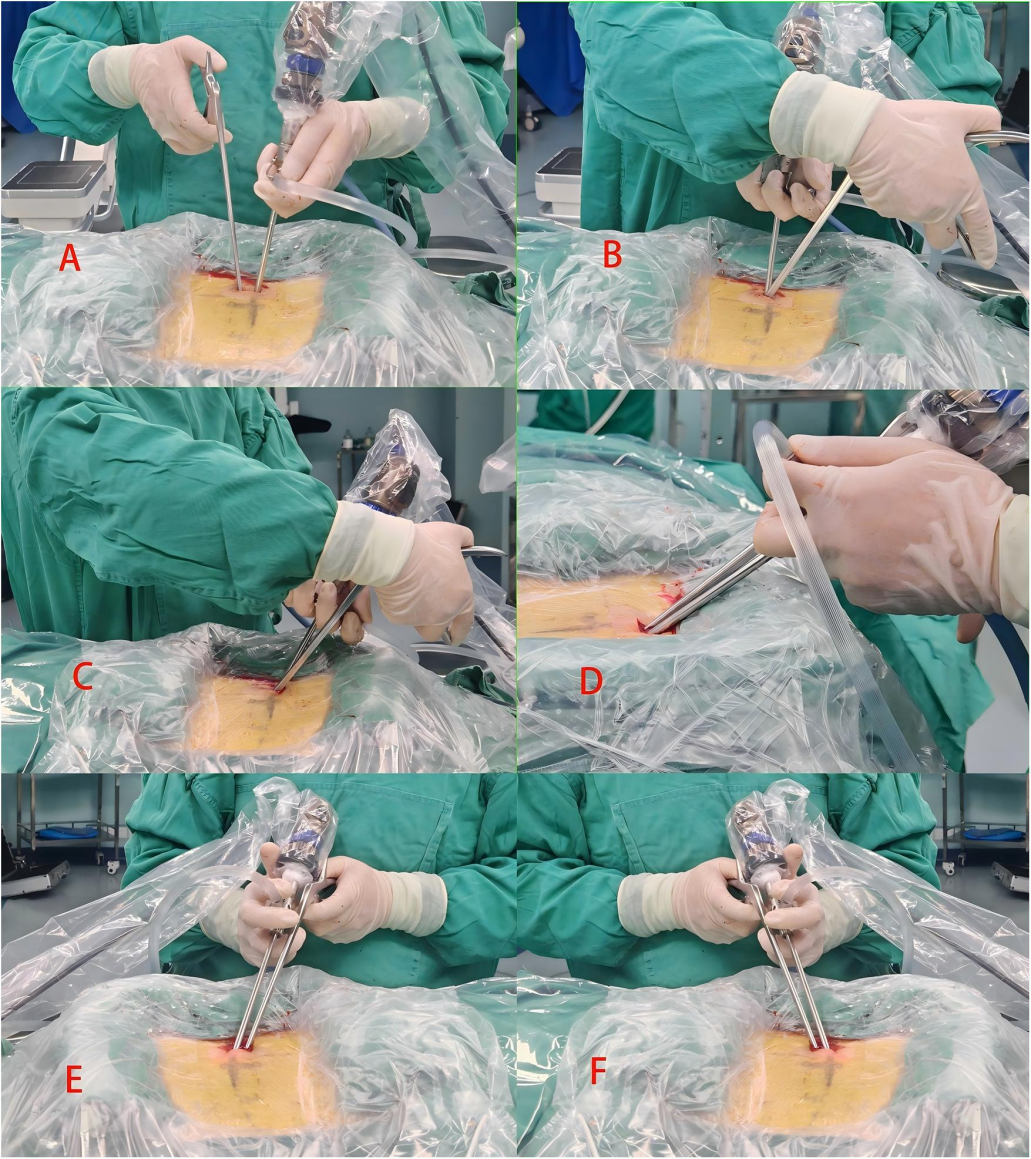
“Endoscopic Hybrid Collaborative Manipulation Technique”: **(A)** triangular operation; **(B–D)** non-coaxial rotation; **(E,F)** coaxial translation and swing.

After general anesthesia, the patient was placed in the prone position, and a C-arm fluoroscope was used to localize the target level. The center point was defined as the intersection of the line connecting the midpoints of the vertebral pedicles of the target segment and the intervertebral space. A longitudinal incision of 1.5–2.5 cm was made at this point (Figures 1, 2), and sequential dilation was performed to establish a single portal that integrated both working and observation channels. Thorough decompression was then carried out according to the modified “circle-drawing” ULBD technique (Figures 3, 4): (1) a radiofrequency probe was used to remove soft tissues in the intervertebral space, exposing the lamina and the attachment of the ligamentum flavum; (2) a high-speed burr or osteotome was used to remove part of the lamina and completely detach the hypertrophic ligamentum flavum; (3) a nerve dissector was used to assess nerve root mobility and, if necessary, to enlarge the contralateral lateral recess; (4) adequate decompression of the dural sac was confirmed under continuous irrigation with normal saline.

#### 2.2.2 UBE technique

According to standardized guidelines (15), the center was defined as the intersection of the line parallel to the base of the spinous process at the target level and the line connecting the medial borders of the pedicles. A viewing incision (approximately 1 cm) and a working incision (approximately 1.5 cm) were made approximately 1.5 cm above and below this intersection (Figures 1, 2). Under endoscopic guidance, bony structures were resected, and nerve roots were decompressed using a lamina punch and pituitary forceps. An aqueous medium was used to maintain a clear visual field throughout the procedure. The decompression technique was otherwise identical to that described for UNSES.

### 2.3 Assessment indicators and data collection

#### 2.3.1 Perioperative indicators

Surgical efficiency: total operative time (from skin incision to suture), postoperative hemoglobin decrease (estimated by the difference between preoperative and postoperative hemoglobin levels); minimally invasive evaluation: total incision length, number of intraoperative fluoroscopy exposures.

Recovery parameters: length of postoperative hospital stay, complication rate.

#### 2.3.2 Functional assessment system

Clinical outcomes were evaluated preoperatively and at 3 and 12 months postoperatively using the Visual Analog Scale (VAS, 0–10) for low back and leg pain, the Japanese Orthopaedic Association (JOA) score (0–29) for neurological function (16), and the Oswestry Disability Index (ODI, 0%–100%) for disability and quality of life (17).

#### 2.3.3 Imaging analysis

**Figure 5:**
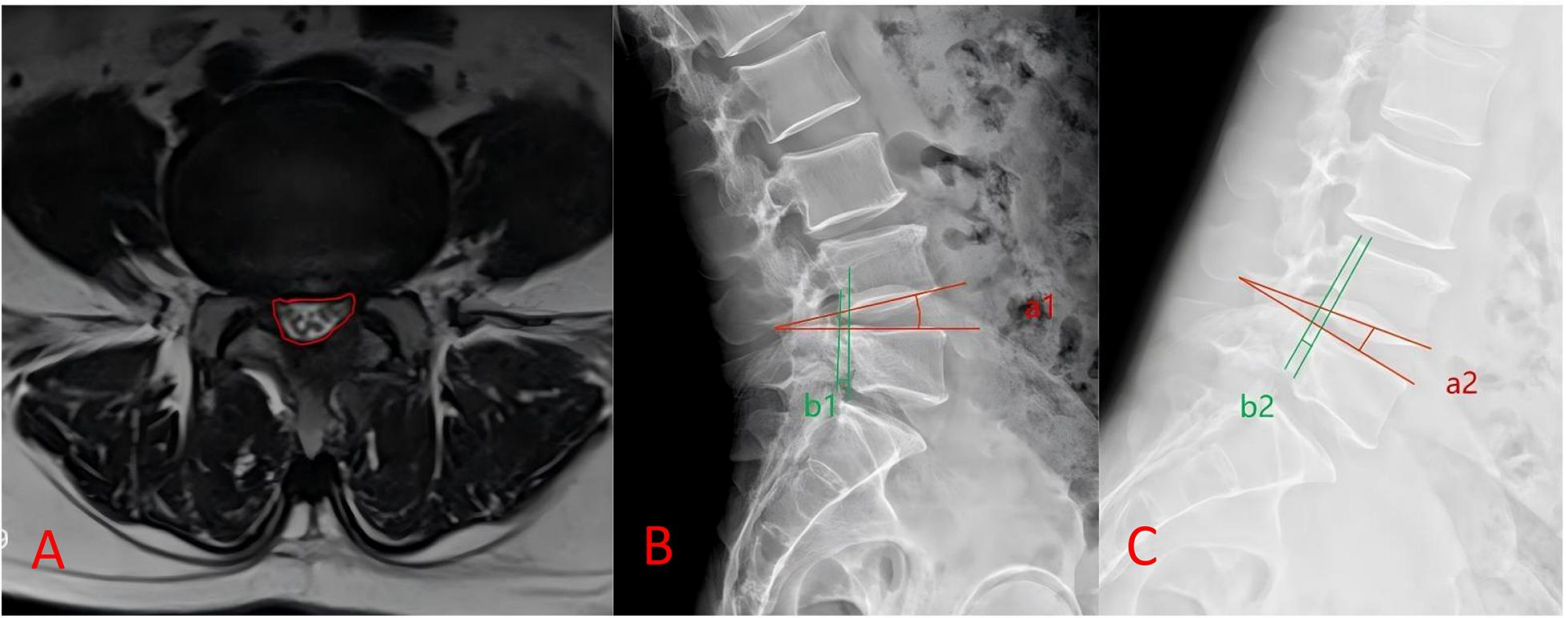
Schematic diagram of imaging measurements. **(A)** Dural sac cross-sectional area (DCSA) measurements; **(B,C)** responsible segmental (L4/5) range of motion (ROM) and responsible segmental sagittal displacement (ST) measurements in hyperextension and hyperflexion positions, L4/5 ROM = a1–a2; L4/5 ST = b1–b2.

Lumbar spine MRI was performed at 12 months postoperatively to measure the dural sac cross-sectional area (DCSA), which was calculated at the narrowest point on the axial view of the symptomatic level. Dynamic stability parameters, including lumbar range of motion (ROM) and sagittal translation (ST), were assessed using flexion-extension radiography at 12 months postoperatively. Two blinded observers independently performed all radiographic measurements, and the mean values were used for analysis (Figure 5).

### 2.4 Statistical analysis

SPSS 26.0 was used for data analysis. Measurement data conforming to a normal distribution were expressed as mean ± standard deviation (SD), and intergroup comparisons were performed using the independent-samples *t*-test. For non-normal data, the Mann–Whitney *U* test was used. Two-way repeated-measures ANOVA was used to analyze repeated-measures data (e.g., VAS scores), and comparisons of categorical variables were made using the *χ*^2^ test or Fisher's exact probability method. A significance threshold of *α* = 0.05 was set.

## 3 Results

Tables 1–3 show that the two groups were well balanced, with no statistically significant differences in age, sex distribution, symptom duration, distribution of stenotic segments, or type of surgical procedure (all *P* > 0.05).

**Table 1:** Baseline characteristics of the two groups.

Parameters	UNSES group (*n* = 96)	UBE group (*n* = 99)	Test statistic	*P* value
Age (year)	68.3 ± 7.2	67.9 ± 6.8	*t* = 0.32	0.752
Female (%)	58.3%	60.6%	*χ*^2^ = 0.15	0.694
Duration of symptoms (months)	18.4 ± 6.2	17.9 ± 5.7	*t* = 0.63	0.634

**Table 2:** Distribution of stenotic segments.

Groups	L3/4	L4/5	L5/S1	Total	Test statistic	*P* value
UNSES	22	46	28	96	*χ*^2^ = 0.644	0.724
UBE	21	53	25	99		

**Table 3:** Decompression surgery.

Groups	ULBD alone	ULBD with discectomy	Total	Test statistic	*P* value
UNSES	68	28	96	*χ*^2^ = 0.548	0.532
UBE	66	33	99		

The UNSES group was associated with shorter operative time (65.2 ± 8.1 vs. 72.5 ± 9.3 min, *P* < 0.001), less hemoglobin decrease (6.2 ± 1.4 vs. 6.8 ± 2.5 g/L, *P* = 0.039), smaller incision length (1.8 ± 0.3 vs. 2.5 ± 0.4 cm, *P* < 0.001), and fewer fluoroscopy exposures (3.1 ± 1.2 vs. 4.5 ± 2.0, *P* < 0.001) than the UBE group. The mean postoperative hospital stay was 4.2 ± 1.1 days in the UNSES group and 4.5 ± 1.3 days in the UBE group, with no statistically significant difference (*P* = 0.118; Table 4).

**Table 4:** Perioperative results.

Parameters	UNSES group (*n* = 96)	UBE group (*n* = 99)	Test statistic	*P* value
Surgical time (minutes)	65.2 ± 8.1	72.5 ± 9.3	*t* = −6.32	<0.001
Hemoglobin decrease (g/L)	6.2 ± 1.4	6.8 ± 2.5	*t* = −2.08	0.039
Fluoroscopy exposures (times)	3.1 ± 1.2	4.5 ± 2.0	*U* = 2,850	<0.001
Length of stay (days)	4.2 ± 1.1	4.5 ± 1.3	*t* = −1.56	0.118
Incision length (cm)	1.8 ± 0.3	2.5 ± 0.4	*t* = −14.22	<0.001

Compared with preoperative values, all functional scores (VAS, JOA, ODI) showed postoperative improvement at each postoperative time point in both groups (*P* < 0.05). No between-group differences were observed in VAS, JOA, or ODI scores at any time point (*P* > 0.05; Table 5).

**Table 5:** Clinical recovery trajectory.

Parameters	UNSES group (*n* = 96)	UBE group (*n* = 99)	Test statistic	*P* value
Preoperative VAS (lumbar)	4.5 ± 1.2	4.7 ± 1.0	−1.26	0.208
Postoperative VAS (lumbar, 3 months)	2.4 ± 1.0	2.5 ± 1.0	−0.69	0.492
Postoperative VAS (lumbar, 12 months)	2.0 ± 0.9	1.9 ± 0.9	0.76	0.447
Preoperative VAS (leg)	7.8 ± 1.1	7.6 ± 1.2	1.22	0.224
Postoperative VAS (leg, 3 months)	2.3 ± 0.7	2.4 ± 1.0	−0.82	0.415
Postoperative VAS (leg, 12 months)	1.9 ± 1.0	1.7 ± 1.3	1.22	0.224
Preoperative ODI (%)	64.4 ± 12.3	66.5 ± 11.9	−1.22	0.225
Postoperative ODI (3 months)	24.7 ± 5.1	23.8 ± 6.1	1.15	0.252
Postoperative ODI (12 months)	18.3 ± 5.2	19.1 ± 5.7	−1.05	0.295
Preoperative JOA score	12.1 ± 2.0	12.4 ± 1.9	−1.08	0.281
Postoperative JOA score (3 months)	22.5 ± 3.5	23.1 ± 3.4	−1.18	0.239
Postoperative JOA score (12 months)	23.8 ± 3.6	23.5 ± 3.7	0.53	0.595

* The *P* values shown correspond to intergroup comparisons (UNSES vs. UBE). All postoperative values were significantly improved compared with preoperative values (*P* < 0.05, data not shown in table).

**Figure 6:**
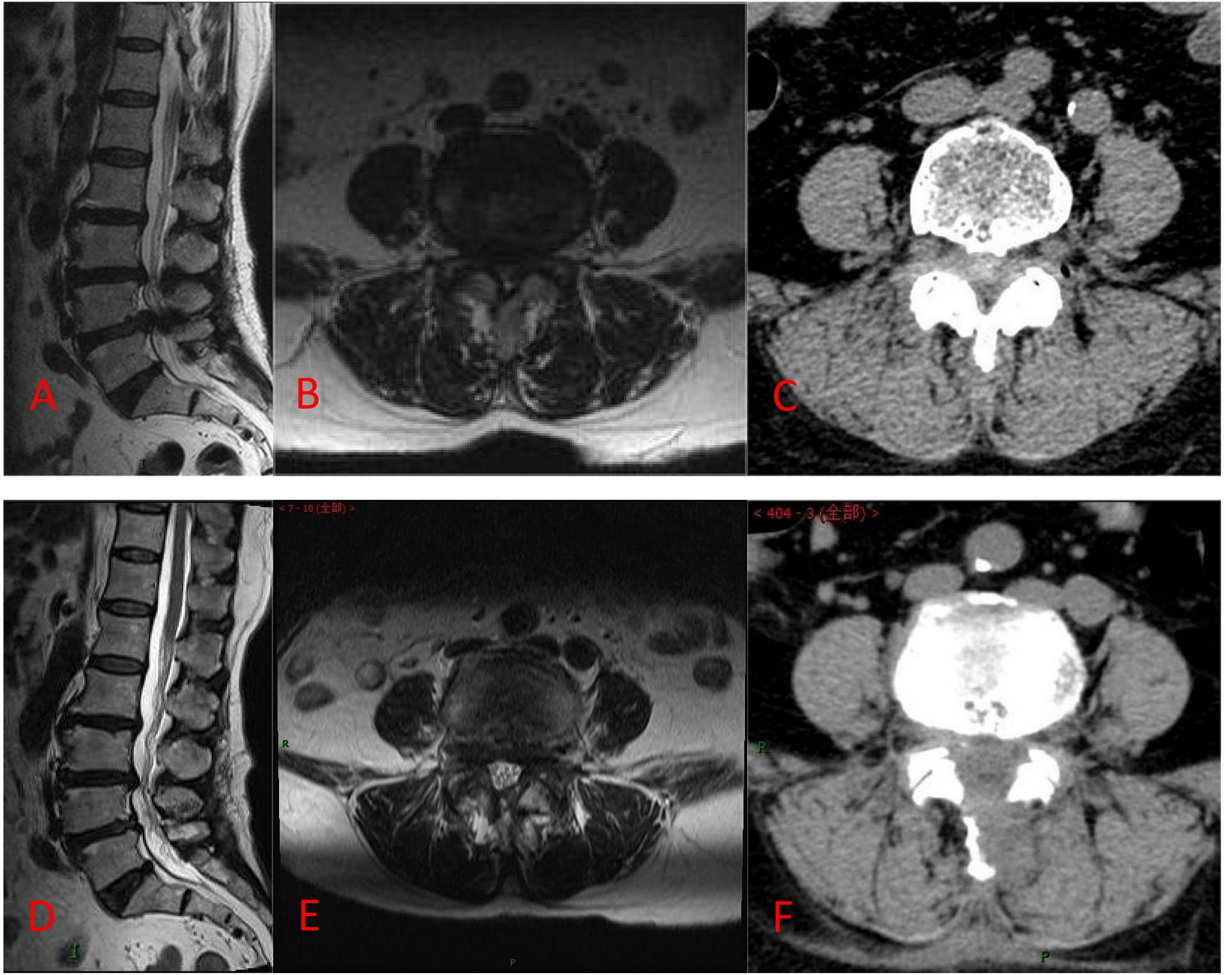
Images from a patient treated with the UNSES technique. **(A–C)** Preoperative MRI and CT show a disc herniation with spinal canal stenosis at the L4–5 segment; **(D–F)** postoperative MRI and CT show the disc herniation was excised and the spinal canal was adequately decompressed.

At 12 months postoperatively, DCSA increased from baseline in both groups (*P* < 0.05), whereas ROM and ST remained largely unchanged. No between-group differences were observed in DCSA, ROM, or ST at any time point (*P* > 0.05; Table 6). Representative radiographic images are shown in Figure 6.

**Table 6:** Radiographic outcomes.

Parameters	UNSES group (*n* = 96)	UBE group (*n* = 99)	Test statistic	*P* value
Preoperative DCSA (mm^2^)	92.5 ± 18.7	89.8 ± 17.2	1.19	0.236
Postoperative 12 months DCSA (mm^2^)	168.3 ± 24.5	166.7 ± 22.8	0.51	0.613
Preoperative ROM (°)	8.5 ± 1.5	8.4 ± 1.7	0.43	0.668
Postoperative 12 months ROM (°)	8.6 ± 1.7	8.5 ± 1.8	0.38	0.705
Preoperative ST (mm)	1.4 ± 0.5	1.3 ± 0.6	1.34	0.182
Postoperative 12 months ST (mm)	1.5 ± 0.4	1.4 ± 0.5	1.58	0.116

* The *P* values shown correspond to intergroup comparisons (UNSES vs. UBE). Postoperative DCSA was significantly increased compared with preoperative values in both groups (*P* < 0.05, data not shown in table). No significant changes were observed in ROM or ST between preoperative and postoperative measurements in either group (*P* > 0.05, data not shown in table).

The incidence rates of dural tear, transient paresthesia, surgical site infection, and reoperation were numerically lower in the UNSES group than in the UBE group; however, none of these differences reached statistical significance (*P* > 0.05; Table 7).

**Table 7:** Safety profile (complications and reoperation rates).

Parameters	UNSES group (*n* = 96)	UBE group (*n* = 99)	Test statistic	*P* value
Dural tear rate	1 (1.04%)	4 (4.04%)	–	0.198
Transient paresthesia rate	2 (2.08%)	3 (3.03%)	–	0.674
Surgical site infection rate	0 (0%)	2 (2.02%)	–	0.497
Reoperation rate	2 (2.08%)	3 (3.03%)	–	0.674

* Fisher's exact test.

## 4 Discussion

The present study compared UNSES and UBE for the treatment of single-segment lumbar spinal stenosis. The main findings were that both techniques were associated with postoperative improvement in pain and functional outcomes, and with dural sac decompression at 12 months, with no between-group differences in clinical or radiographic outcomes during follow-up. By contrast, differences between the two procedures were observed in several perioperative parameters, including operative time, hemoglobin decrease, incision length, and fluoroscopy exposure. These findings suggest that UNSES and UBE may provide comparable mid-term effectiveness, while differing in certain procedural characteristics.

At 12 months postoperatively, no between-group differences were observed in pain relief or functional recovery, and both groups showed postoperative improvement compared with baseline. This finding is broadly consistent with previous studies of UBE, which have reported clinical outcomes comparable to those of microscopic or open decompression for lumbar spinal stenosis, particularly in terms of pain relief and functional improvement (18, 19). In the present study, UNSES was associated with clinical outcomes at 12 months that were similar to those of UBE. Taken together, these findings suggest that when adequate decompression is achieved, different endoscopic decompression strategies may provide comparable mid-term clinical benefit in patients with single-segment LSS.

Although the two techniques showed comparable clinical and radiographic outcomes at 12 months, differences were observed in several perioperative parameters. Compared with UBE, UNSES was associated with shorter operative time, less hemoglobin decrease, a smaller incision length, and fewer fluoroscopy exposures. These differences may be related to the technical characteristics of UNSES. First, the single-incision configuration may simplify localization and eliminate the need to establish and maintain two separate portals, thereby reducing fluoroscopy use and improving procedural efficiency. Second, the hybrid synergistic approach allows the viewing and working instruments to function independently within the same portal, which may facilitate hand coordination while preserving a flexible working angle. Third, the non-coaxial rotation technique may expand the effective working space without requiring an additional incision, thereby allowing decompression through a limited soft-tissue corridor. In addition, unlike UBE, in which inadequate irrigation outflow may occasionally compromise visualization and require further channel adjustment, UNSES may help maintain procedural continuity by allowing the endoscope and instruments to retract the fascia directly, thereby preserving unobstructed outflow. Nevertheless, these perioperative differences were modest in magnitude and were not accompanied by shorter hospital stay or lower complication rates; therefore, they should be interpreted cautiously rather than as evidence of overall clinical superiority.

Radiographic findings in the present study provide further context for interpreting the clinical results. At 12 months, DCSA increased in both groups, indicating that adequate decompression had been achieved. At the same time, ROM and ST remained largely unchanged, and no between-group differences were observed. These findings suggest that neither technique was associated with radiographic evidence of instability during follow-up. Because excessive resection of the facet joints may adversely affect segmental biomechanics (20, 21), the absence of measurable deterioration in ROM or ST in our cohort may reflect adequate decompression without substantial disruption of stabilizing structures. However, the follow-up duration was limited to 12 months, and these results should not be interpreted as definitive evidence regarding long-term lumbar stability.

Regarding safety, both techniques showed low complication rates, and no statistically significant between-group differences were observed. Although the rates of dural tear, transient paresthesia, surgical site infection, and reoperation were numerically lower in the UNSES group, these differences did not reach statistical significance and should not be overinterpreted. In our cohort, dural tears were mainly associated with adhesions between the ligamentum flavum and the dural sac, underscoring the importance of careful tissue handling and clear identification of the dissection plane during decompression. From a practical perspective, the present findings suggest that the choice between UNSES and UBE should not be based solely on small differences in perioperative parameters, but should also take into account surgeon experience, familiarity with the technique, and the specific operative setting. In other words, the procedural convenience observed in this study for UNSES may be clinically relevant, but it does not, by itself, establish overall superiority over UBE.

This study has several limitations. First, its retrospective, non-randomized design introduces the possibility of selection bias and residual confounding, despite the two groups' comparable baseline characteristics. Second, all procedures were performed by a single experienced surgeon. Although this reduces inter-operator variability, it may also limit the generalizability of the findings to other surgeons and institutions. Third, the 12-month follow-up period is relatively short for evaluating long-term durability and delayed postoperative instability; therefore, the present study cannot determine whether the observed similarities in radiographic stability will persist over time. In addition, although surgeon experience was standardized to the extent possible, this study was not designed to directly compare the learning curves of UNSES and UBE. Finally, radiographic measurements were performed manually, and some degree of measurement variability cannot be excluded. Future multicenter, prospective studies with longer follow-up, more standardized reporting of surgeon-related factors, and more objective imaging assessment methods are needed to better define the respective roles of UNSES and UBE in the treatment of LSS.

## 5 Conclusion

In summary, for the treatment of single-segment lumbar spinal stenosis, both UNSES and UBE were associated with improvement in clinical symptoms and functional status, and with spinal canal decompression at 1-year follow-up, with similarly low complication rates. Compared with UBE, UNSES was associated with differences in several perioperative parameters, including operative time, hemoglobin decrease, incision length, and fluoroscopy exposure. Given the retrospective design and relatively short follow-up period, these findings should be interpreted with caution. Further multicenter, prospective, randomized controlled studies with longer follow-up are needed to confirm the durability of these outcomes and to clarify the respective roles of UNSES and UBE in the treatment of LSS.

## Data Availability

The raw data supporting the conclusions of this article will be made available by the authors, without undue reservation.
